# Payn on Physic

**Published:** 1892-03-05

**Authors:** 


### THE DOCTOR'S ARMCHAIR.

PAYN ON PHYSIC.

MR. JAMES PAYN has tabled a protest in the *New York Independent* against what he calls the turning of the surgeon's bull's-eye upon literary men. Certain American physicians, it appears, have taken the lead in this policeman's duty. They have set forth the proposition that "literary workers should take a light breakfast, eat nothing at noon, and enjoy an evening meal when the work of the day is quite done." Mr. Payn thinks he has those American physicians on the hip, and sets to work to belabour them with the club of experience—not of medical experience, be it observed—but of literary. "There is nothing," he says, "in which men's minds vary so much as in the conditions they find favourable for literary composition. Some write like Thackeray by fits and starts, some like Trollope, with the regularity of clockwork, some by day, some by night, some fasting, some exhilarated by gin and water (like Byron), some stimulated by tobacco (like Tennyson); there is no sort of rule for general guidance."

All this is most true, but it does not prove anything, and we can quite believe that Mr. Payn never intended that it should. It is fine literary protoplasm, but it does not rise to the dignity of a back-bone, or even of a gelatinous notochord. The American physicians lay down a general rule for literary men as a class. Mr. Payn replies by giving us four or five names that represent almost a century of literature, the owners of which did not conform to that rule, and who appear to have done very well without it. But let Mr. Payn apply this method of argumentation to another department of human activity, "Thou shalt not steal," said Moses, and a great many decidedly rational people have endorsed that elementary proposition. But some few distinguished persons, such, for example, as Barabbas, Paul Jones, Richard Turpin, and sundry American and European financiers, have been as oblivious of Moses' inconvenient suggestion as if it had never been made, and yet they and a great many others have come to no immediately apparent harm by the process. But is it, therefore, desirable to steal?

Mr. Payn is an able and an amiable man. One cannot but be sorry for him in that he wraps himself up so completely in the mantle of the specialist. As a writer he is in many respects admirable. But if he is so praiseworthy when he neglects the plainly obvious rules of simple and non-pedantic science, how much more entirely excellent might he have been if he had practised a little docility towards concrete physiological facts throughout his life! Mr. Walter Besant has recorded the opinion that a man of letters should walk seven or eight miles a day, and have frequent changes of scene. Mr. Payn holds up his hands in horror. "If I was to walk eight miles," says he, "I should certainly be driven home, and in a hearse. As to 'frequent changes,' when I want a 'scene' I go and look at it; but I should as soon think of taking a railway journey 'anywhere' as of undergoing a surgical operation when there was nothing the matter."

Now this little utterance reveals to the physiologist who happens to be familiar with, and to take pleasure in Mr. Payn's literary work, something more perhaps than he himself quite intended. A man who cannot walk eight miles must be either very old or very feeble. Mr. Payn is not old; he must, therefore, be feeble. And, indeed, one of the most striking characteristics of his literary work is its want of nerve, firmness, and grip. It is extremely pleasant, and so is a ripe gooseberry. But no one would select a ripe gooseberry as an emblem of strength and endurance. It is quite possible that Mr. Payn has no ambition for strong effectiveness or immortality in literature. But this very want of ambition to make work strong and permanently effective is a conspicuous mark by which the physiologist diagnoses weakness—weakness not only of body, but of everything pertaining to the body; weakness of every kind of tissue, brain and nerve tissue among the rest.

A man who is weak may be satisfied with his weakness; but that does not convert the weakness into strength, or make it a less unfortunate endowment. A scribe with a first-rate ambition may be only a second or third rate writer; but a scribe with a second or third rate ambition must inevitably be a second or third rate writer, or even of a lower rank than that. There can be nothing more entirely fitting than that a writer should produce the best that is possible for him, under the most favourable circumstances. As a rule, the most favourable circumstances are quite essential for the production of the best. One of the most indispensable of all favourable conditions is sound, genial, and wholesome health; and for sound, genial, and wholesome health a certain amount of physical exercise and an adequate quantity of fresh air, with sound sleep and an easy digestion, are conditions that can by no possibility be dispensed with. It is absolutely nothing to the purpose to tell us that Thackeray wrote by fits and starts, and that Byron was in his most productive mood when drinking gin and water. As a matter of fact, Byron produced a great quantity of immature, and a greater quantity of very unworthy stuff. He was a man of rare genius; and if he had been as wholesome and as noble in intellectual quality as he was splendidly brilliant, he might have ranked with Goethe, Shakespeare, and Milton, instead of being, as Carlyle might have called him, a mere ineffectuality and flash in the pan.

The truth is that Mr. James Payn, and others like him, are survivals from an outworn period. The very elements of real science—that is of practical, as distinguished from book science—seem to be unknown to them. From a Darwinian point of view, they belong to what may be called a primitive and natural, as distinguished from a scientifically-educated and developed literary period. All their methods of working are primitive and untaught. They are like the farmer who ploughs with a couple of bullocks and a wooden ploughshare instead of turning over the soil of his fields with a steam cultivator. The idea of stirring up one's brain with gin and water, or of alternately working at ten o'clock in the morning, or after a heavy dinner, or at midnight, as the primitive unregulated impulse of the moment may suggest, is simply barbarous. It is the method of the old-fashioned and half-educated Nonconformist parson, who, after visiting and gossiping about all the week, sat down to prepare his Sunday morning's sermon after his Saturday night's nine o'clock supper.

This kind of thing, indeed, will not do at all. Mr. Payn's methods may last Mr. Payn's life, but they will be simply fatal to the younger generation. Not fatal merely from the bodily health point of view, but also from the intellectual and literary. The young writer of to-day is in the market of open competition. If he desires to live permanently by literature he must lay his plans for producing work of a constantly improving quality. No man can produce high-class work with low-class tools. What is the instrument with which the literary man works? It is his brain; and in exact proportion as that brain is in excellent order will it produce excellent work. Mr. Payn, and others like him, may protest against this dictum until Doomsday: but the facts are against them. And the facts always have carried, and always will carry the day in spite of everything that can be said or done to the contrary. Mr. James Payn affects to smile at the utterances of the American physicians. He would be much wiser, and it would be much better for him, if he would try to understand the inner essence of what they teach and so far as is possible, to carry it out in his habits and daily life.

